# Supplementary material for: Examining the effectiveness of consuming flour made from agronomically biofortified wheat (Zincol-2016/NR-421) for improving Zn status in women in a low-resource setting in Pakistan: study protocol for a randomised, double-blind, controlled cross-over trial (BiZiFED)
Source: BMJ Open. 2018 Apr 17;8(4):e021364. doi: 10.1136/bmjopen-2017-021364 (PMC5905768; doi:10.1136/bmjopen-2017-021364)
Supplement: Author's manuscript [file bmjopen-2017-021364.draft_revisions.pdf]

BMJ Open

BMJ Open is committed to open peer review. As part of this commitment we make the peer review history of every article we publish publicly available.

When an article is published we post the peer reviewers' comments and the authors' responses online. We also post the versions of the paper that were used during peer review. These are the versions that the peer review comments apply to.

The versions of the paper that follow are the versions that were submitted during the peer review process. They are not the versions of record or the final published versions. They should not be cited or distributed as the published version of this manuscript.

BMJ Open is an open access journal and the full, final, typeset and author-corrected version of record of the manuscript is available on our site with no access controls, subscription charges or pay-per-view fees (http://bmjopen.bmj.com).

If you have any questions on BMJ Open's open peer review process please email info.bmjopen@bmj.com

BMJ Open

Examining the effectiveness of consuming flour made from agronomically biofortified wheat (Zincol-2016/NR-421) for improving Zn status in women in a low resource setting Pakistan: Study protocol for a randomised, double blind, controlled cross over trial (BiZiFED).

Journal:	*BMJ Open*
Manuscript ID	bmjopen-2017-021364
Article Type:	Protocol
Date Submitted by the Author:	02-Jan-2018

Complete List of Authors:	Lowe, Nicola; University of Central Lancashire, International Institute of Nutritional Sciences and Applied Food Safety Studies, Faculty of Health and Wellbeing<br>Khan, Muhammad ; Khyber Medical University, Institute of Basic Medical sciences<br>Broadley, Martin; University of Nottingham, Sutton Bonnington Campus<br>Zia, Munir; Fauji Fertilizer Co Ltd<br>McArdle, H; University of Aberdeen; University of Nottingham School of Health Sciences<br>Joy, Edward; London School of Hygiene and Tropical Medicine, Department of Population Health<br>Ohly, Heather; University of Central Lancashire, International Institute of Nutritional Sciences and Applied Food Safety Studies, Faculty of Health and Wellbeing<br>Shahzad, Babar ; Khyber Medical University, Institute of Basic Medical Sciences<br>Ullah, Ubaid; Khyber Medical University, Institute of Basic Medical Sciences<br>Kabana, Gul; Abaseen Foundation<br>Medhi, Rashid; Abaseen Foundation<br>Afridi, Mukhtiar; Abaseen Foundation; Lady Reading Hospital, Division of Medicine, Medical Teaching Institution

Keywords:	zinc, wheat, Pakistan, agronomic biofortification, zinc status

SCHOLARONE™
Manuscripts

Title: Examining the effectiveness of consuming flour made from agronomically biofortified wheat (Zincol-2016/NR-421) for improving Zn status in women in a low resource setting Pakistan: Study protocol for a randomised, participant blind, controlled cross over trial (BiZiFED).

Authors

Corresponding Author: Nicola M. Lowe. International Institute of Nutritional Sciences and Applied Food Safety Studies, Faculty of Health and Wellbeing, University of Central Lancashire,UK. PR1 2HE. United Kingdom.

Email: nmlowe@uclan.ac.uk

Telephone: 01772 893599

Co-Authors:

Muhammad Jaffar Khan. Institute of Basic Medical sciences, Khyber Medical University Peshawar Pakistan 25000Martin R. Broadley. School of Biosciences, University of Nottingham, Sutton Bonington Campus, Leicestershire, LE12 5RD, UK.

Munir H. Zia. Research & Development Department, Fauji Fertilizer Company Limited, 156-The Mall, Rawalpindi, Pakistan.

Harry J McArdle, School of Biosciences, University of Nottingham, Sutton Bonnington Campus, Leicestershire, LE12 5RD, UK.

Edward J.M. Joy, Department of Population Health, London School of Hygiene & Tropical Medicine, London, WC1E 7HT, UK.

Heather Ohly International Institute of Nutritional Sciences and Applied Food Safety Studies, Faculty of Health and Wellbeing, University of Central Lancashire,UK.

Babar Shahzad. Institute of Basic Medical sciences, Khyber Medical University Peshawar Pakistan 25000.

Ubaid Ullah. Institute of Basic Medical sciences, Khyber Medical University Peshawar Pakistan 25000

Gul Kabana. Abaseen Foundation, Peshawar, Pakistan

Rashid Medhi. Abaseen Foundation, Peshawar, Pakistan

Mukhtiar Zaman Chief Executive Abaseen Foundation Pakistan & Professor Division of Medicine Medical Teaching Institution Lady Reading Hospital, Peshawar, Pakistan

Key Words: zinc, wheat, agronomic biofortification, Pakistan, zinc status

Word Count: 4062

For peer review only

Title: Examining the effectiveness of consuming flour made from agronomically biofortified wheat (Zincol-2016/NR-421) for improving Zn status in women in a low resource setting Pakistan: Study protocol for a randomised, double blind, controlled cross over trial (BiZiFED).

Abstract

Introduction: Dietary zinc (Zn) deficiency is a global problem, particularly in low and middle-income countries where access to rich, animal-source foods of Zn is limited due to poverty. In Pakistan, Zn deficiency affects over 40% of the adult female population, resulting in sub-optimal immune status and increased likelihood of complications during pregnancy.

Methods and analysis: We are conducting a double blind, randomised controlled feeding study with crossover design, in a low resource setting in Pakistan. Households are provided with flour milled from genetically and agronomically biofortified grain (Zincol-2016/NR-421) or control grain (Galaxy-2013. Fifty households have been recruited. Each household includes a female aged 16-49 years who is neither pregnant nor breastfeeding, and not currently consuming nutritional supplements. These women are the primary study participants. All households were provided with control flour for an initial 2-week baseline period, followed by an 8-week intervention period where 25 households receive biofortified flour (Group A) and 25 households receive control flour (Group B). After this eight-week period, Group A and B cross over, receiving control and biofortified flour respectively for eight weeks. Tissue (blood, hair and nails) will be collected from the women at five time points: baseline, mid and end of period 1, mid and end of period 2.

Ethics and dissemination: Ethical approval has been granted from the lead University (reference number: STEMH 697 FR) and the collaborating institution in Pakistan. The final study methods (including any modifications) will be published in peer reviewed journals, alongside the study outcomes on completion of the data analysis. In addition, findings will be disseminated to the scientific community via conference presentations and abstracts and communicated to the study participants through the village elders at an appropriate community forum.

Registration details: The trial has been registered with the ISRCTN registry, study ID ISRCTN83678069.

Strengths and limitations of the study:

1) The project is the first feeding study to combine a high-Zn variety of wheat (Zincol-2016/NR-421) with a high grain Zn concentration with the application of Zn fertilizers to maximise the Zn content of wheat for flour production;

For peer review only

For peer review only - http://bmjopen.bmj.com/site/about/guidelines.xhtml

2) The study is the first randomised controlled trial to determine the effectiveness of consuming flour from the Zincol-2016/NR-421 strain of wheat on Zn status in women of child-bearing age;

3) The study will evaluate the use of novel biomarkers of Zn status in low resource settings;

4) A limitation of this study is the relatively small sample size (n=50). This is compensated for by the cross over design which strengthens the statistical power.

Introduction

Dietary zinc (Zn) deficiency is a global problem, affecting 17% of the world's population, with the greatest burden in Low and Middle Income Countries (LMICs) [2 3]. The most recent national survey in Pakistan indicates that over 40% of women are Zn deficient [4]. The consequences of Zn deficiency are profound and far-reaching, ranging from increased individual morbidity and mortality to problems with community and regional economic development [5].

Attempts to alleviate the problem of Zn deficiency, both in Pakistan and in other countries, have followed several strategies. Providing micronutrient supplements, usually using "sprinkles", has been attempted under different circumstances, generally with some success [6]. However, it is an expensive and intensive approach, and without close supervision it can often be that the intended target, for example a pregnant woman, does not end up receiving the supplement. Fortification of foods at the processing stage has also been widely adopted, but there are significant problems with compliance and efficacy. A major issue is that poorer households in rural areas of developing countries typically grow the majority of their food and process it at village or community level. Thus, cereal fortification at large-scale processing facilities may not reach poorer households, where micronutrient deficiencies are often more prevalent and severe.

An alternative approach is to intervene at agricultural production stage through biofortification to achieve greater concentrations of bioavailable vitamins or elements in the edible portion of crops [7]. Biofortification can be realised through crop breeding or application of element-enriched fertilisers, known as "agronomic biofortification", which is particularly important in Pakistan where plant-available soil Zn concentrations are low [8]. Such Zn deficient soils that constitute 52% at national scale are also prevalent in Peshawar, Khyber Pakhtunkhwa Province, where plant-available soil Zn concentrations are significantly below the threshold 1 mg/kg in 75% of the soils analysed. Farmers in Pakistan rarely apply Zn fertilizers to their crops except for rice. Biofortification approaches have focused on staple

For peer review only - http://bmjopen.bmj.com/site/about/guidelines.xhtml

crops because they are widely consumed including households with low purchasing power. Recently, varieties of maize, pearl millet and beans with high iron (Fe) and Zn concentrations have been developed by the HarvestPlus programme for deployment in low-income settings. Human feeding studies have demonstrated that such varieties can lead to improvements in Fe and Zn intake and status [9,10], although there may be issues with low bioavailability [11]. Agronomic biofortification has not previously been deployed in a low-income setting but does have policy precedent in Finland, where granular fertilizers were enriched with selenium (Se) since the mid 1980's with consequent increases in human dietary Se intakes and eventual elimination of Se deficiency as a public health problem [12].

Preliminary studies have shown that Zn concentrations in wheat can be raised substantially through biofortification strategies[13]. It is notable that increasing levels of nutrients in the whole plant is not sufficient; elements must be translocated to the edible component of the grain (endosperm) and must be bioavailable. Phytic acid (PA) is the most important inhibitor of Zn absorption from wheat, therefore the Zn:PA molar ratio of the endosperm is an important consideration. The process of element accumulation in the endosperm is not simple. For example, Zn moves through many different transport processes prior to being deposited in the seed [14]. In a recent set of experiments within the HarvestPlus program over a period of eight years, scientists in Pakistan using traditional breeding techniques have been able to generate a "high Zn" variety of wheat that is adapted to Pakistan conditions. The variety (Zincol-2016/NR-421) contains more Zn per kg of grain compared to traditional varieties. However, grain Zn concentration will also depend on the availability of Zn in the soil, and there are likely to be synergistic effects of breeding for high Zn with application of Zn fertilizers.

The efficacy of increasing dietary intake through biofortification on individual and population Zn status is notoriously difficult to measure. The assessment of Zn status has been the subject of considerable research and is summarised comprehensively in two recent reviews supported by multinational expert groups [15 16]. Both reviews conclude that plasma or serum Zn concentration, the most widely used biomarker, is useful provided that various confounding factors are considered (including concurrent infection and inflammation). However, the plasma/serum must be collected following stringent methodology to avoid contamination and can be difficult to achieve in field settings. Hair Zn concentration is also potentially useful, and samples are easy to collect and store. In addition, emerging techniques, including the measurement of DNA fragmentation using the Comet assay and analysis of nail Zn concentration using laser ablation, are gaining credibility as useful biomarkers of Zn status. However, field trials are needed to evaluate their usefulness.

For peer review only - http://bmjopen.bmj.com/site/about/guidelines.xhtml

The primary aim of this study is to measure the impact of consuming flour made from the recently released high Zn wheat grain variety Zincol-2016/NR-421 on dietary Zn intake and biomarkers of Zn status, in a low resource rural community setting in Pakistan. The secondary aim is to evaluate the potential usefulness of new biomarkers of Zn status. These aims will be achieved through conducting a randomised controlled feeding study with a crossover design. Comparison of outcome measures will be made following consumption of flour made from the biofortified wheat variety, Zincol-2016/NR-421, and the control, non-biofortified variety, Galaxy. The trial has been registered with the ISRCTN registry, study ID ISRCTN83678069 [1].

METHODS AND ANALYSIS

Study Setting and recruitment.

This study is being conducted from the Health Centre that serves a community of approximately 5000 households in an area of 8 km^2 on the outskirts of Peshawar in Khyber Pakhtunkhwa (KPK). This community was chosen because previous studies have indicated that Zn deficiency is widespread, the diet is limited and primarily vegetable-based (thus low in bioavailable Zn) and the staple is wheat which is used for making chapatti and roti[17]. The catchment area, known as a Union Council, is made up of 10 villages (clusters). Out of 10 clusters in the union council, 5 were randomly selected-, from which equal number of households were randomly selected for the study. Selection of these clusters and households into intervention or control arm was done using a block design. Water samples were collected in duplicate from four different communal tube wells (hand pump) supplying each village, and analysed for mineral content by inductively coupled plasma-mass spectrometry (ICP-MS). The mean Zn concentration of the water from one cluster was high enough to significantly impact on daily Zn intake (>250 μg/L) therefore this cluster was eliminated from the study and an alternative cluster was randomly selected. The mean Zn concentration in the water analysed from the five included clusters was ≤120 μg/L and therefore not considered to add significantly to the daily Zn intake, based on a consumption of 2-4 L water per day (<0.5 mg Zn per day).

Ten households from each cluster was randomly selected. The head of the household was approached, the purpose of the study explained and, if eligible, the family invited to participate in the trial. If the head of the household declined, another house from the cluster was randomly selected and the invitation process repeated. Inclusion criteria were: the household included a female aged 16-49 years who was neither pregnant nor breastfeeding, and not currently consuming nutritional supplements. When there was more than 1 eligible woman in the household, one was conveniently selected with agreement from the head of

For peer review only - http://bmjopen.bmj.com/site/about/guidelines.xhtml

the houeshold. There were no additional exclusion criteria. Following informed consent, each household was assigned to treatment arm A or B (refer to figure 1) using a block design. A sample size of 40, 95% significance level (2-sided) and 90% power, will enable an increase in plasma Zn concentration of 3.1 µg/dL to be detected. The target was to recruit 50 households (50 eligible women) to be able to accommodate a 20% attrition rate during the 18-week study. The project manager led the recruitment process because he is known to the community through previous health-related projects associated with the Abaseen Foundation PK (AFPK), a local NGO and key member of this project team, along with its sister organisation Abaseen Foundation UK (AFUK). There is a high level of trust and cooperation between community members and the AFPK who operate the Health Centre that serves this Union Council, therefore high levels of participant enrolment has been achieved. The project manager was responsible for allocating participants to each arm of the crossover trial using a block design. Only the project manager had access to this information.

The participants and the remaining research team members, including the PI and CoPIs, database manager and data analysts were blind to the order in which households received the biofortified or control flour. Unblinding will be permissible if any adverse reactions are reported by the households during the trial.

Study design

We are conducting a randomised controlled feeding study with crossover design in which households are provided with flour milled from genetically and agronomically biofortified grain (Zincol-2016/NR-421) or control grain (Galaxy-2013). Galaxy-2013 was chosen for the control grain because it is widely cultivated across the country and is popular among farmers due to its higher yield. It did not receive Zn-containing fertilizers. Zincol-2016 (formerly known as NR-421 strain/line) was bred by the National Agriculture Research System, Pakistan with the support of HarvestPlus and approved by the Punjab Seed Council, Pakistan in 2016.[18] The grain for both the genotypes was sown at a farm in District Vehari District, Punjab, Pakistan in November 2016, and harvested in May 2017 by our project partner, Fauji Fertilizer Company (FFC) Limited. During the growing period, Zn-enriched fertilizer (Zn 13% as EDTA Zn) was applied to Zincol-2016 genotype at 1.25 kg/ha through injection into the irrigation system. This was followed by five foliar sprays of Zn ($ZnSO_4 \cdot H_2O$ 33% Zn) applied at 1 kg/ha of product dissolved in 250 L of water and sprayed per hectare at the booting stage. Analyses revealed that the Zn concentration in grains were 62 (SD 7.7) mg/kg and 27 (SD 4.3) mg/kg for the biofortified and control grain respectively. The grain was transported from the farm to the study field site in clearly labelled sacks (control and biofortified). The bags of grain were manually cleaned (hand removal of debris, stones,

For peer review only - http://bmjopen.bmj.com/site/about/guidelines.xhtml

stalks), mixed and milled at a local facility. Care was taken to avoid cross contamination from previously milled grain. Samples of flour from each sack (top, middle and bottom) were retained for analysis.

The intervention trial is comprised of a 2-week baseline equilibration period and two 8-week intervention/control periods. The baseline period started on 1st October 2017. During the baseline period, all the participating households were provided with control flour. After two weeks, Group A received biofortified flour and Group B received control flour. After 8 weeks, the two groups will cross over, with Group A receiving control flour and Group B receiving biofortified flour for the final 8 weeks (Figure 1). The flour will be provided on a fortnightly basis for consumption by the entire household (average seven individuals, including children). Families will be asked not to consume any other flour within the household during the 18-week study period. Compliance will be monitored at each flour distribution time point through interview with the head of the household. The number of meals consumed outside the household by the primary study participant (woman of child- bearing age) will be recorded. Our previous knowledge of this community indicates that, other than community events such as weddings, it is unusual for women to eat outside the home.

Figure 1: Overview of Objective 1

Primary outcome measures

Flour is used primarily for making roti and chapatti in this community. The contribution of the total Zn intake from foods made from flour will be determined using 24-hour recall, coupled with food composition databases (e.g. WinDiets 2005® Research software, Robert Gordon University, UK). In addition, a selection of composite meal samples will be analysed. The impact of consuming the biofortified flour on the Zn status of women of childbearing age will be assessed by measuring plasma Zn concentration on ICP-MS analysis, DNA fragmentation by comet assay, and hair and nail Zn concentration by ICP-MS. In addition, we will measure concentrations of inflammatory biomarkers C-reactive protein (CRP) and alpha 1-acid glycoprotein that will be used to adjust plasma Zn concentration if required [19].

Dietary Analysis

Dietary nutrient intake will be assessed by 24-hour recall, conducted by interviewing the women participants by the study Nutritionist who has local knowledge of the foods consumed and speaks Pushto, the local language. From our previous research[17] we know that the local diet is very limited in variety and monotonous and therefore a total of five recall interviews, taken at each of the sampling points (Figure 1), will give a good estimate of

For peer review only - http://bmjopen.bmj.com/site/about/guidelines.xhtml

typical nutrient intake. Portion sizes will be estimated using household measures, using items that are familiar to the households (e.g. plates, cups, spoons). Macro and micronutrient composition, plus phytate will be calculated from appropriate food composition databases. In addition, a selection of commonly consumed composite meals will be analysed for mineral, macronutrient and phytic acid concentration. During this interview, the nutritionist will also collect any data regarding adverse health-related events since the last measurement time point. Mothers will also be asked to report on the frequency and duration of diarrhoeal episodes amongst children in the household. Any major concerns will be reported to the Health Centre Doctor for follow up.

Biomarkers of Zn status

Tissue samples will be collected from participants attending the Health Centre at five time points: baseline, mid and end of intervention phase 1 and mid and end of phase 2 (Figure 1).

Plasma Zn concentration

Whole blood will be collected into trace element free tubes (BD Diagnostics, Switzerland) and the plasma separated by centrifugation within 40 minutes. Plasma 300 µl aliquots will be transferred into Eppendorf tubes and stored at -20°C. Once the trial is complete, plasma samples will be shipped on dry ice to the University of Nottingham for analysis by ICP-MS.

DNA fragmentation analysis

DNA fragmentation (measured using the Comet assay) has been shown to decrease following dietary Zn supplementation [20]. Whole blood will be collected by venous blood draw. Duplicate samples of 50 µl of whole blood will be mixed with 50 µl cryopreservation buffer (85% RPMI, 15% DMSO) in a cryovial, and stored in a cryopreservation container in a fridge for up to 8 hours. Samples will be transferred from the health centre to KMU on ice and placed in a -80°C freezer overnight (up to 12 hours), then transferred to liquid nitrogen for storage until samples at all five time points have been collected. DNA fragmentation will be quantified using the method of Singh et al [21 22], and Comet Assay IV Lite software (Perceptive Instruments, London, UK) for tail moment measurement.

Nail and hair analysis

Whole hairs will be collected by combing through the hair and collecting individual hairs from the comb, making sure that the tag (cells from follicle) is attached to the proximal end of the hair. Nail samples will be collected at baseline. Nail samples will be collected two more times at approximately 7 months (average time taken for a nail to grow from formation in the nail bed to the tip of the finger) from the start of phase 1 and phase 2. The Zn concentration

For peer review only - http://bmjopen.bmj.com/site/about/guidelines.xhtml

of hair and nail samples will be determined using laser induced breakdown spectroscopy[23] at NeurobioTex (Galveston, Texas, USA).

Additional outcomes

Anthropometry

Height and weight of the women was measured at baseline, and weight will be monitored at each sample collection time point. Height was measured using a height board. The participant was to stand still with her back towards the scale, with the heels, back of the calves, upper back and the back of their head touching the board with the scale. The head was positioned so that the Frankfurt line was straight. The measuring device was lowered gently on the hair until it rested gently on the scalp. For the measurement of weight, women were asked to relax for 5 minutes before being asked to remove added clothes, shoes, socks, anything in the pocket, watch or necklace. The woman was asked to stand on the marked position on the scale until the digital reading or the reading needle stabilized. For consistency, weight was measured three times and the average calculated. In addition, the height and weight of at least one child under ten years in each household will be monitored throughout the intervention period. Mothers will also be asked to keep a diary of the frequency and duration of diarrhoeal episodes of children in the household throughout the intervention period.

Haematological and Micronutrient analyses

Haematocrit, haemoglobin, mean corpuscular volume (MCV), total iron binding capacity (TIBC), transferrin saturation will be estimated using clinical haematology methods. Blood samples will be analysed for micronutrient status including serum transferrin receptor and ferritin (sandwich ELISA), Fe and Se concentrations (ICP-MS), vitamin A (retinol binding protein (RBP)), and inflammatory markers (α1-acid glycoprotein (AGP) and C- reactive protein (CRP)).

Dark adaptation

The potential impact of increasing dietary Zn intake on dark adaptation was measured at baseline week 2, week 10 and week 18 during the participants' visits to the Health Centre. Dark adaptation was measured using a novel device developed by Palmer and co-workers[24].

Stool samples for parasite infection and microbiome analysis

Stools were collected at baseline week 2, week 10 and week 18. Samples will be analysed within 24 hours for presence of parasites. Samples will be stored to create a biobank for future short chain fatty acid and microbiome analysis.

For peer review only - http://bmjopen.bmj.com/site/about/guidelines.xhtml

DATA COLLECTION AND STORAGE

Sample analysis will take place in a number of different specialist laboratories in the UK (University of Nottingham: plasma Zn, Fe and Se, water, flour), USA (NeurobioTex: nail and hair Zn concentration) and Pakistan (Khyber Medical University: comet assay, haematology, serum transferring receptor, ferritin, RBP, and inflammatory markers). Comet assay tail moment analysis will be undertaken by both PK (KMU) and UK (University of Central Lancashire) based teams, and the results compared. Any significant differences will be resolved with expert support from the Children's Hospital Oakland Research Institute (CHORI). Sample analysis will be undertaken in duplicate as a minimum requirement. Data entry will be undertaken by the expert laboratory, and encrypted spreadsheets sent to the data hub where a final check for anomalies will be made by the database manager. Any potential anomalies identified will be checked with the expert lab. All data will be managed compliant with standard BBSRC policies http://www.bbsrc.ac.uk/web/FILES/Policies/data-sharing-policy.pdf. The University of Central Lancashire (UCLan) will be the data hub for the project; UCLan adheres to an Open Data policy and has an open data repository. Metadata will be collected in the form of "read me" files using basic Dublin Core. This study will generate small quantities of personal data from the analysis of blood and hair/nail samples. Most of the participants will be illiterate so verbal informed consent will be obtained and recorded by the project manager. Participants will be given an ID number on recruitment, and this will be used in all data files. The list of names associated with the ID numbers will be known only by the project manager, and will be stored in a locked filing cabinet. Data collected in the field on laptops will be password protected. UCLan systems are automatically backed up. Transfer of data between laboratories will be in the form of encrypted excel or word files. Data and metadata will be securely stored in a project database and reported on publication. Data will be shared with the project steering committee during the bi-monthly meetings. This committee is comprised of the PI and Co-Is. The database manager will undertake the role of data monitoring and all members will have access to the final database.

STATISTICAL ANALYSIS PLAN

The analysis will be performed as intention to treat. Descriptive statistics will summarise participants' characteristics and outcome measurements at each time point, using methods appropriate for the type of data. Most study outcome measurements will be quantitative data. The primary outcome, plasma Zn concentration, will be collected at 5 time points (baseline, week 4, 8, 12 and 16). After adjustment for inflammatory markers and as described above, the primary outcome will be assessed in two type comparisons:

For peer review only - http://bmjopen.bmj.com/site/about/guidelines.xhtml

I. Within group (A and B) comparisons.

Within each group of participants, the study outcome will be compared at appropriate time point (baseline will be compared with week 4 and week 8, week 4 compared with week 8, week 12 with week 16) to assess trend of the change (increase or decrease) in Zn concentrations. The pairwise comparisons will be done using paired t-test or Wilcoxon signed rank test if normality assumptions for t-test are not satisfied. Repeated measures ANOVA (or its non-parametric analogue) will be applied for comparisons at multiple time points (e.g. to compare study outcome at baseline, week 4 and week 8).

II. Comparisons between group A and B.

To answer the primary research question of determining the effectiveness of consuming flour made from agronomically biofortified wheat for improving Zn status, study outcomes will be compared between group A and B at week 8 and week 16, complemented with comparisons at interim collection points, week 4 and week 12. The pairwise comparisons will be done using independent samples t-test or Mann-Whitney U test as appropriate. Linear mixed modelling will be used for adjusted analyses to account for the baseline, repeated measurements and relevant covariates. Similar analysis will be done for all secondary outcomes measuring Zn status.

ETHICS AND DISSEMINATION

Ethical approval for the research has been granted from the lead University (UCLan) (unique reference number: STEMH 697 FR) and the collaborating institution in Pakistan, Khyber Medical University. All data collected will be anonymized and data protection protocols followed when information is shared between participating institutions.

The final study methods (including any modifications) will be published in peer reviewed journals, alongside the study outcomes on completion of the data analysis. Authorship will be restricted to those who have contributed to the design of the study, data collection and analysis, in accordance with journal guidelines. In addition to peer reviewed journals, findings will be disseminated to the scientific community via conference presentations and abstracts. The findings of the study will be communicated to the study participants through the village elders at an appropriate community forum. More widely, the AFPK can access some of the most difficult to reach communities in KPK and the Federally Administered Tribal Area (FATA), including through tribal networks, local Jirga and religious leaders. We will work with our contacts in the agriculture sector to exchange knowledge with key actors. For example, the Agricultural Services Department of FFC provides soil, water, fertilizer, and

For peer review only

plant analysis and recommendation services (in addition to other farmer education/training programmes) to the farming community throughout the country. Project partners and collaborators are well connected to ensure that new evidence can support policy.

AUTHORS CONTRIBUTIONS

Nicola Lowe, Martin Broadley, Munir Zia, Harry McArdle, Jaffar Khan, Edward Joy, and Mukhtiar Zaman conceived the research questions and designed the study protocol. Nicola Lowe is the principal investigator, Martin Broadley, Jaffar Khan, Harry McArdle, Edward Joy, Munir Zia, and Mukhtiar Zaman are co-investigators. Rashid Medhi is managing the community engagement and recruitment, Babar Shahzad, Ubaid Ullah and Gul Khubana are conducting the field work and sample collection. Heather Ohly was involved in the original conception of the study, and is managing the study database at the project hub (UCLan). All authors have read and approved this manuscript.

FUNDING

This work is supported by BBSRC Global Challenges Research Fund, Foundation Awards for Global Agriculture and Food Systems Research. Grant Number BB/P02338X/1

We are grateful for the in-kind contribution of Fauji Fertilizer Company (FFC) Limited for crop management and production, and to HarvestPlus for supplying the Zincol-2016/NR-421 wheat grain for sowing.

We would also like to acknowledge the in-kind contribution of Professor Janet King and Dr. Swapna Shenvi at the Children's Hospital Oakland Research Institute (CHORI) for support with the Comet assay, and Dr Amanda Palmer and Dr Katie Healy at Johns Hopkins University for training and loan of the dark adaptation detection equipment.

The authors gratefully acknowledge the Abaseen Foundation, Pakistan for use of the Health Centre and facilitating access to the community. Also Dr Sveltlana Tishkovskaya at UCLan for Statistical support.

CONFLICTS OF INTEREST

The authors report no conflicts of interest.

REFERENCES

1. ISRCTN83678069. Biofortification with zinc in flour for eliminating deficiency: BioMed Central; 2017 [Available from: https://www.isrctn.com/ISRCTN83678069 accessed 30/11/2017].

For peer review only - http://bmjopen.bmj.com/site/about/guidelines.xhtml

2. Wessells KR, Brown KH. Estimating the Global Prevalence of Zinc Deficiency: Results Based on Zinc Availability in National Food Supplies and the Prevalence of Stunting. *Plos One* 2012;7(11)

3. Kumssa DB, Joy EJM, Ander EL, et al. Dietary calcium and zinc deficiency risks are decreasing but remain prevalent. *Sci Rep-Uk* 2015;5

4. Agha Khan University. Planning Commission. Planning and Development Division. Government of Pakistan. National Nutrition Survey Pakistan 2011.

5. Boerma JT, Bryce J, et al. Mind the gap: equity and trends in coverage of maternal, newborn, and child health services in 54 Countdown countries. *Lancet* 2008;371(9620):1259-67.

6. Allen LH, Peerson JM, Olney DK. Provision of multiple rather than two or fewer micronutrients more effectively improves growth and other outcomes in micronutrient-deficient children and adults. *J Nutr* 2009;139(5):1022-30.

7. Varo P, Alfthan G, Ekholm P, et al. Selenium intake and serum selenium in Finland: effects of soil fertilization with selenium. *Am J Clin Nutr* 1988;48(2):324-9.

8. Edward J. M. Joy WA, Munir H. Zia, Diriba B. Kumssa, Scott D. Young, E. Louise Ander, Michael J. Watts, Alexander J. Stein, Martin R. Broadley. Valuing increased zinc (Zn) fertiliser-use in Pakistan. *Plant and Soil* 2016.

9. Cercamondi CI, Egli IM, Mitchikpe E, et al. Total iron absorption by young women from iron-biofortified pearl millet composite meals is double that from regular millet meals but less than that from post-harvest iron-fortified millet meals. *J Nutr* 2013;143(9):1376-82.

10. Chomba E, Westcott CM, Westcott JE, et al. Zinc absorption from biofortified maize meets the requirements of young rural Zambian children. *The Journal of nutrition* 2015;145(3):514-9.

11. Petry N, Egli I, Gahutu JB, et al. Stable iron isotope studies in Rwandese women indicate that the common bean has limited potential as a vehicle for iron biofortification. *J Nutr* 2012;142(3):492-7.

12. Alfthan G, Eurola M, Ekholm P, et al. Effects of nationwide addition of selenium to fertilizers on foods, and animal and human health in Finland: From deficiency to optimal selenium status of the population. *JTEMB* 2015;31:142-47.

13. Coralie Signorell DM, Christophe Zeder, Fabian Tay, Erick Boy, Neelam Khetarpaul, Ismail Cakmak, Michael Zimmermann. Evaluation of Zinc Bioavailability in Humans from Foliar Zinc Biofortified Wheat and from Intrinsic vs. Extrinsic Zn Labels in Biofortified Wheat. *Eur J Nutr & Food Safety* 2015;5(5):863-64.

14. Olsen LI, Palmgren MG. Many rivers to cross: the journey of zinc from soil to seed. *Frontiers in PLand Sci* 2014;5:1-6.

For peer review only

For peer review only - http://bmjopen.bmj.com/site/about/guidelines.xhtml

15. King JC, Gibson RS, Krebs NF, Lowe NM, Siekmann JH, Raiten DJ. Biomarkers of Nutrition for Development (BOND)-Zinc Review. *J Nutr* 2016.

16. Lowe NM, Fekete K, Decsi T. Methods of assessment of zinc status in humans: a systematic review. *Am J Clin Nutr* 2009;89(6):2040s-51s.

17. Khan MJ, Usama UU, Lowe N, Broadley M, Afridi MZ, Zia M, McArdle HM, Young S. Effect of agronomically biofortified zinc flour on zinc and selenium status in resource poor settings; a randomised control trial. *Proc Nutr Soc* 2017;76(OCE4).

18. Singh RaG, Velu. Zinc-Biofortified Wheat: Harnessing Genetic Diversity for Improved Nutritional Quality. *Science Brief: Biofortification No 1 (May 2017) CIMMYT, HarvestPlus, and the Global Crop Diversity Trust Bonn, Germany*.

19. Raiten DJ, Sakr Ashour FA, Ross AC, et al. Inflammation and Nutritional Science for Programs/Policies and Interpretation of Research Evidence (INSPIRE). *J Nutr* 2015;145(5):1039S-108S.

20. Joray ML, Yu T-W, Ho E, et al. Zinc supplementation reduced DNA breaks in Ethiopian women. *Nutr Res* 2015;35(1):49-55.

21. Singh NP, McCoy MT, Tice RR, et al. A simple technique for quantitation of low levels of DNA damage in individual cells. *Exp Cell Res* 1988;175(1):184-91.

22. Zyba SJ, Shenvi SV, Killilea DW, et al. A moderate increase in dietary zinc reduces DNA strand breaks in leukocytes and alters plasma proteins without changing plasma zinc concentrations. *Am J Clin Nutr* 2017;105(2):343-51.

23. Riberdy VA, Frederickson CJ, Rehse SJ. Determination of the Zinc Concentration in Human Fingernails Using Laser-Induced Breakdown Spectroscopy. *App Spectr* 2017;71(4):567-82.

24. Labrique AB, Palmer AC, Healy K, et al. A novel device for assessing dark adaptation in field settings. *BMC Ophthalmol* 2015;15:74.

For peer review only - http://bmjopen.bmj.com/site/about/guidelines.xhtml

442

443

444

445

446

447

448

Figure Legend

Figure 1: Schematic overview of objective 1, randomised controlled feeding study with crossover design.

For peer review only

For peer review only - http://bmjopen.bmj.com/site/about/guidelines.xhtml

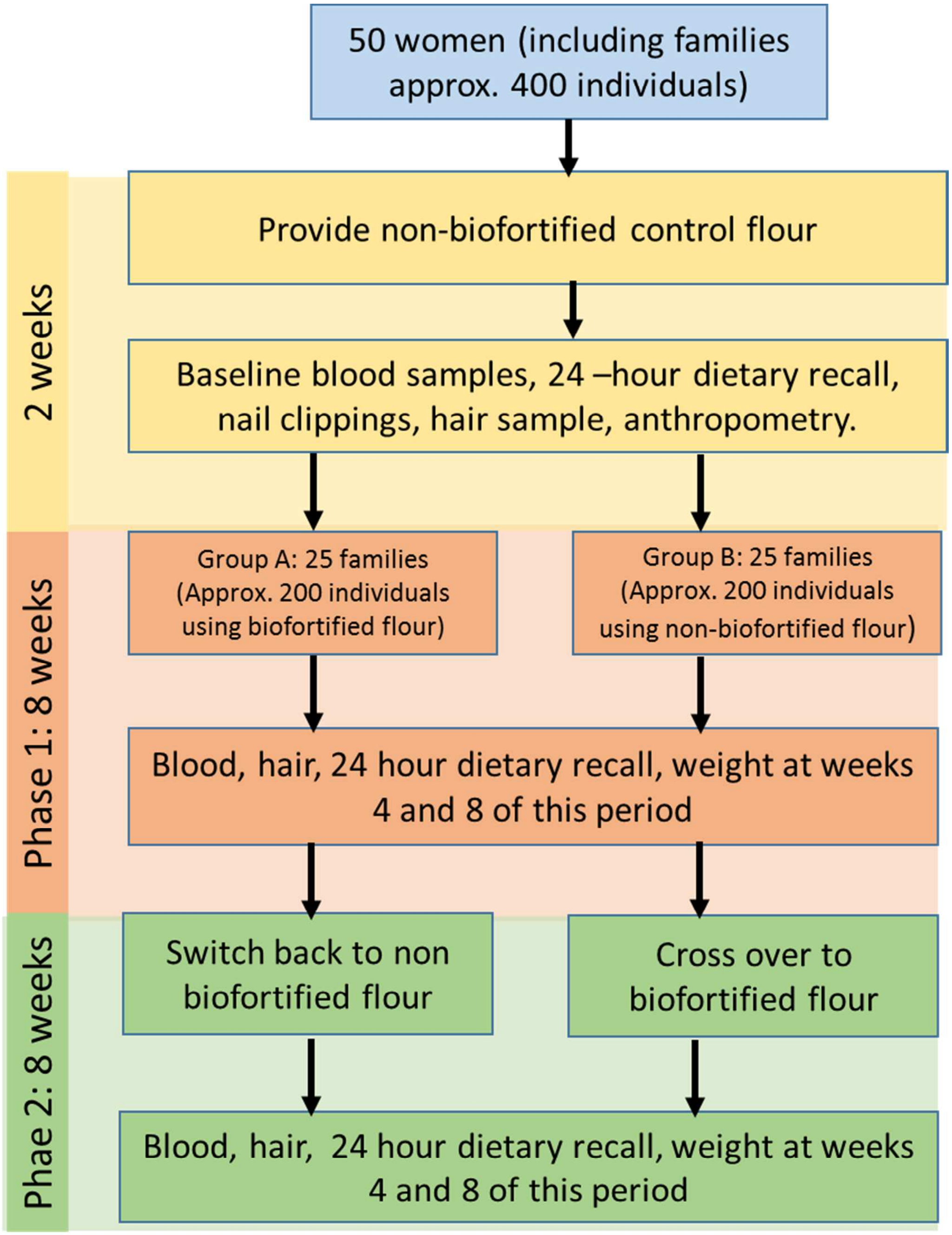
50 women (including families approx. 400 individuals)
2 weeks
Provide non-biofortified control flour
Baseline blood samples, 24 –hour dietary recall, nail clippings, hair sample, anthropometry.
Phase 1: 8 weeks
Group A: 25 families (Approx. 200 individuals using biofortified flour)
Group B: 25 families (Approx. 200 individuals using non-biofortified flour)
Blood, hair, 24 hour dietary recall, weight at weeks 4 and 8 of this period
Phae 2: 8 weeks
Switch back to non biofortified flour
Cross over to biofortified flour
Blood, hair, 24 hour dietary recall, weight at weeks 4 and 8 of this period

STANDARD PROTOCOL ITEMS: RECOMMENDATIONS FOR INTERVENTIONAL TRIALS

SPIRIT 2013 Checklist: Recommended items to address in a clinical trial protocol and related documents*

Section/item	Item No	Description	Addressed on page number
**Administrative information**			
Title	1	Descriptive title identifying the study design, population, interventions, and, if applicable, trial acronym	_____1________
Trial registration	2a	Trial identifier and registry name. If not yet registered, name of intended registry	_____1________
	2b	All items from the World Health Organization Trial Registration Data Set	___yes_______
Protocol version	3	Date and version identifier	_______N/A___
Funding	4	Sources and types of financial, material, and other support	____11_____
Roles and responsibilities	5a	Names, affiliations, and roles of protocol contributors	12 & cover page
	5b	Name and contact information for the trial sponsor	____N/A_____
	5c	Role of study sponsor and funders, if any, in study design; collection, management, analysis, and interpretation of data; writing of the report; and the decision to submit the report for publication, including whether they will have ultimate authority over any of these activities	_____N/A_______
	5d	Composition, roles, and responsibilities of the coordinating centre, steering committee, endpoint adjudication committee, data management team, and other individuals or groups overseeing the trial, if applicable (see Item 21a for data monitoring committee)	___12____

For peer review only

For peer review only - http://bmjopen.bmj.com/site/about/guidelines.xhtml

**Introduction**			
Background and rationale	6a	Description of research question and justification for undertaking the trial, including summary of relevant studies (published and unpublished) examining benefits and harms for each intervention	__2-4__
	6b	Explanation for choice of comparators	__5_______
Objectives	7	Specific objectives or hypotheses	______4_______
Trial design	8	Description of trial design including type of trial (eg, parallel group, crossover, factorial, single group), allocation ratio, and framework (eg, superiority, equivalence, noninferiority, exploratory)	__5-7______
**Methods: Participants, interventions, and outcomes**			
Study setting	9	Description of study settings (eg, community clinic, academic hospital) and list of countries where data will be collected. Reference to where list of study sites can be obtained	__4,5_____
Eligibility criteria	10	Inclusion and exclusion criteria for participants. If applicable, eligibility criteria for study centres and individuals who will perform the interventions (eg, surgeons, psychotherapists)	_4,5___
Interventions	11a	Interventions for each group with sufficient detail to allow replication, including how and when they will be administered	__4,5_________
	11b	Criteria for discontinuing or modifying allocated interventions for a given trial participant (eg, drug dose change in response to harms, participant request, or improving/worsening disease)	_____N/A______
	11c	Strategies to improve adherence to intervention protocols, and any procedures for monitoring adherence (eg, drug tablet return, laboratory tests)	____6_______
	11d	Relevant concomitant care and interventions that are permitted or prohibited during the trial	___N/A______
Outcomes	12	Primary, secondary, and other outcomes, including the specific measurement variable (eg, systolic blood pressure), analysis metric (eg, change from baseline, final value, time to event), method of aggregation (eg, median, proportion), and time point for each outcome. Explanation of the clinical relevance of chosen efficacy and harm outcomes is strongly recommended	____7-9______
Participant timeline	13	Time schedule of enrolment, interventions (including any run-ins and washouts), assessments, and visits for participants. A schematic diagram is highly recommended (see Figure)	_7 (FIGURE 1)

For peer review only - http://bmjopen.bmj.com/site/about/guidelines.xhtml

Sample size	14	Estimated number of participants needed to achieve study objectives and how it was determined, including clinical and statistical assumptions supporting any sample size calculations	___5__________
Recruitment	15	Strategies for achieving adequate participant enrolment to reach target sample size	____4________

Methods: Assignment of interventions (for controlled trials)

Allocation:			
Sequence generation	16a	Method of generating the allocation sequence (eg, computer-generated random numbers), and list of any factors for stratification. To reduce predictability of a random sequence, details of any planned restriction (eg, blocking) should be provided in a separate document that is unavailable to those who enrol participants or assign interventions	_____N/A_______ _
Allocation concealment mechanism	16b	Mechanism of implementing the allocation sequence (eg, central telephone; sequentially numbered, opaque, sealed envelopes), describing any steps to conceal the sequence until interventions are assigned	____N/A_______
Implementation	16c	Who will generate the allocation sequence, who will enrol participants, and who will assign participants to interventions	____5_______
Blinding (masking)	17a	Who will be blinded after assignment to interventions (eg, trial participants, care providers, outcome assessors, data analysts), and how	____5________
	17b	If blinded, circumstances under which unblinding is permissible, and procedure for revealing a participant's allocated intervention during the trial	___5_______

Methods: Data collection, management, and analysis

Data collection methods	18a	Plans for assessment and collection of outcome, baseline, and other trial data, including any related processes to promote data quality (eg, duplicate measurements, training of assessors) and a description of study instruments (eg, questionnaires, laboratory tests) along with their reliability and validity, if known. Reference to where data collection forms can be found, if not in the protocol	___7-10______
	18b	Plans to promote participant retention and complete follow-up, including list of any outcome data to be collected for participants who discontinue or deviate from intervention protocols	___10______

For peer review only - http://bmjopen.bmj.com/site/about/guidelines.xhtml

Data management	19	Plans for data entry, coding, security, and storage, including any related processes to promote data quality (eg, double data entry; range checks for data values). Reference to where details of data management procedures can be found, if not in the protocol	___10_______
Statistical methods	20a	Statistical methods for analysing primary and secondary outcomes. Reference to where other details of the statistical analysis plan can be found, if not in the protocol	__10-11____
	20b	Methods for any additional analyses (eg, subgroup and adjusted analyses)	____N/A______
	20c	Definition of analysis population relating to protocol non-adherence (eg, as randomised analysis), and any statistical methods to handle missing data (eg, multiple imputation)	__N/A______
**Methods: Monitoring**			
Data monitoring	21a	Composition of data monitoring committee (DMC); summary of its role and reporting structure; statement of whether it is independent from the sponsor and competing interests; and reference to where further details about its charter can be found, if not in the protocol. Alternatively, an explanation of why a DMC is not needed	_____10_____
	21b	Description of any interim analyses and stopping guidelines, including who will have access to these interim results and make the final decision to terminate the trial	___N/A______
Harms	22	Plans for collecting, assessing, reporting, and managing solicited and spontaneously reported adverse events and other unintended effects of trial interventions or trial conduct	_______8___
Auditing	23	Frequency and procedures for auditing trial conduct, if any, and whether the process will be independent from investigators and the sponsor	___N/A_______
**Ethics and dissemination**			
Research ethics approval	24	Plans for seeking research ethics committee/institutional review board (REC/IRB) approval	____11_____
Protocol amendments	25	Plans for communicating important protocol modifications (eg, changes to eligibility criteria, outcomes, analyses) to relevant parties (eg, investigators, REC/IRBs, trial participants, trial registries, journals, regulators)	_____11_____

For peer review only - http://bmjopen.bmj.com/site/about/guidelines.xhtml

Consent or assent	26a	Who will obtain informed consent or assent from potential trial participants or authorised surrogates, and how (see Item 32)	____5_______
	26b	Additional consent provisions for collection and use of participant data and biological specimens in ancillary studies, if applicable	___N/A_______
Confidentiality	27	How personal information about potential and enrolled participants will be collected, shared, and maintained in order to protect confidentiality before, during, and after the trial	________10___
Declaration of interests	28	Financial and other competing interests for principal investigators for the overall trial and each study site	______12_____
Access to data	29	Statement of who will have access to the final trial dataset, and disclosure of contractual agreements that limit such access for investigators	___10_______
Ancillary and post-trial care	30	Provisions, if any, for ancillary and post-trial care, and for compensation to those who suffer harm from trial participation	__N/A_________
Dissemination policy	31a	Plans for investigators and sponsor to communicate trial results to participants, healthcare professionals, the public, and other relevant groups (eg, via publication, reporting in results databases, or other data sharing arrangements), including any publication restrictions	____11_______
	31b	Authorship eligibility guidelines and any intended use of professional writers	______12_____
	31c	Plans, if any, for granting public access to the full protocol, participant-level dataset, and statistical code	____10_______
**Appendices**			
Informed consent materials	32	Model consent form and other related documentation given to participants and authorised surrogates	_appendix 1 and 2
Biological specimens	33	Plans for collection, laboratory evaluation, and storage of biological specimens for genetic or molecular analysis in the current trial and for future use in ancillary studies, if applicable	_9,10_______

*It is strongly recommended that this checklist be read in conjunction with the SPIRIT 2013 Explanation & Elaboration for important clarification on the items. Amendments to the protocol should be tracked and dated. The SPIRIT checklist is copyrighted by the SPIRIT Group under the Creative Commons "Attribution-NonCommercial-NoDerivs 3.0 Unported" license.

For peer review only - http://bmjopen.bmj.com/site/about/guidelines.xhtml

BMJ Open

Examining the effectiveness of consuming flour made from agronomically biofortified wheat (Zincol-2016/NR-421) for improving Zn status in women in a low resource setting Pakistan: Study protocol for a randomised, double blind, controlled cross over trial (BiZiFED).

Journal:	*BMJ Open*
Manuscript ID	bmjopen-2017-021364.R1
Article Type:	Protocol
Date Submitted by the Author:	16-Feb-2018
Complete List of Authors:	Lowe, Nicola; University of Central Lancashire, International Institute of Nutritional Sciences and Applied Food Safety Studies, Faculty of Health and Wellbeing<br>Khan, Muhammad ; Khyber Medical University, Institute of Basic Medical sciences<br>Broadley, Martin; University of Nottingham, Sutton Bonnington Campus<br>Zia, Munir; Fauji Fertilizer Co Ltd<br>McArdle, H; University of Aberdeen; University of Nottingham School of Health Sciences<br>Joy, Edward; London School of Hygiene and Tropical Medicine, Department of Population Health<br>Ohly, Heather; University of Central Lancashire, International Institute of Nutritional Sciences and Applied Food Safety Studies, Faculty of Health and Wellbeing<br>Shahzad, Babar ; Khyber Medical University, Institute of Basic Medical Sciences<br>Ullah, Ubaid; Khyber Medical University, Institute of Basic Medical Sciences<br>Kabana, Gul; Abaseen Foundation<br>Medhi, Rashid; Abaseen Foundation<br>Afridi, Mukhtiar; Abaseen Foundation; Lady Reading Hospital, Division of Medicine, Medical Teaching Institution
**Primary Subject Heading**:	Global health
Secondary Subject Heading:	Nutrition and metabolism, Public health
Keywords:	zinc, wheat, Pakistan, agronomic biofortification, zinc status

SCHOLARONE™
Manuscripts

Title: Examining the effectiveness of consuming flour made from agronomically biofortified wheat (Zincol-2016/NR-421) for improving Zn status in women in a low resource setting Pakistan: Study protocol for a randomised, double blind, controlled cross over trial (BiZiFED).

Authors

Corresponding Author: Nicola M. Lowe. International Institute of Nutritional Sciences and Applied Food Safety Studies, Faculty of Health and Wellbeing, University of Central Lancashire,UK. PR1 2HE. United Kingdom.

Email: nmlowe@uclan.ac.uk

Telephone: 01772 893599

Co-Authors:

Muhammad Jaffar Khan. Institute of Basic Medical sciences, Khyber Medical University Peshawar Pakistan 25000Martin R. Broadley. School of Biosciences, University of Nottingham, Sutton Bonington Campus, Leicestershire, LE12 5RD, UK.

Munir H. Zia. Research & Development Department, Fauji Fertilizer Company Limited, 156-The Mall, Rawalpindi, Pakistan.

Harry J McArdle, School of Biosciences, University of Nottingham, Sutton Bonnington Campus, Leicestershire, LE12 5RD, UK.

Edward J.M. Joy, Department of Population Health, London School of Hygiene & Tropical Medicine, London, WC1E 7HT, UK.

Heather Ohly International Institute of Nutritional Sciences and Applied Food Safety Studies, Faculty of Health and Wellbeing, University of Central Lancashire,UK.

Babar Shahzad. Institute of Basic Medical sciences, Khyber Medical University Peshawar Pakistan 25000.

Ubaid Ullah. Institute of Basic Medical sciences, Khyber Medical University Peshawar Pakistan 25000

Gul Kabana. Abaseen Foundation, Peshawar, Pakistan

Rashid Medhi. Abaseen Foundation, Peshawar, Pakistan

Mukhtiar Zaman Chief Executive Abaseen Foundation Pakistan & Professor Division of Medicine Medical Teaching Institution Lady Reading Hospital, Peshawar, Pakistan

Key Words: zinc, wheat, agronomic biofortification, Pakistan, zinc status

Word Count: 4062

For peer review only - http://bmjopen.bmj.com/site/about/guidelines.xhtml

Title: Examining the effectiveness of consuming flour made from agronomically biofortified wheat (Zincol-2016/NR-421) for improving Zn status in women in a low resource setting Pakistan: Study protocol for a randomised, double blind, controlled cross over trial (BiZiFED).

Abstract

Introduction: Dietary zinc (Zn) deficiency is a global problem, particularly in low and middle-income countries where access to rich, animal-source foods of Zn is limited due to poverty. In Pakistan, Zn deficiency affects over 40% of the adult female population, resulting in sub-optimal immune status and increased likelihood of complications during pregnancy.

Methods and analysis: We are conducting a double blind, randomised controlled feeding study with crossover design, in a low resource setting in Pakistan. Households were provided with flour milled from genetically and agronomically biofortified grain (Zincol-2016/NR-421) or control grain (Galaxy-2013. Fifty households were recruited. Each household included a female aged 16-49 years who is neither pregnant nor breastfeeding, and not currently consuming nutritional supplements. These women were the primary study participants. All households were provided with control flour for an initial 2-week baseline period, followed by an 8-week intervention period where 25 households receive biofortified flour (Group A) and 25 households receive control flour (Group B). After this eight-week period, Group A and B crossed over, receiving control and biofortified flour respectively for eight weeks. Tissue (blood, hair and nails) have been collected from the women at five time points: baseline, mid and end of period 1, mid and end of period 2.

Ethics and dissemination: Ethical approval was granted from the lead University (reference number: STEMH 697 FR) and the collaborating institution in Pakistan. The final study methods (including any modifications) will be published in peer reviewed journals, alongside the study outcomes on completion of the data analysis. In addition, findings will be disseminated to the scientific community via conference presentations and abstracts and communicated to the study participants through the village elders at an appropriate community forum.

Registration details: The trial has been registered with the ISRCTN registry, study ID ISRCTN83678069.

Strengths and limitations of the study:

1) The study is the first randomised controlled trial to determine the effectiveness of consuming flour from the Zincol-2016/NR-421 strain of wheat on Zn status in women of child-bearing age;

For peer review only - http://bmjopen.bmj.com/site/about/guidelines.xhtml

2) The study will evaluate the use of novel biomarkers of Zn status in low resource settings;

3) A limitation of this study is the relatively small sample size (n=50). This is compensated for by the cross over design which strengthens the statistical power.

Introduction

Dietary zinc (Zn) deficiency is a global problem, affecting 17% of the world's population, with the greatest burden in Low and Middle Income Countries (LMICs) [1, 2]. The most recent national survey in Pakistan indicates that over 40% of women are Zn deficient [3]. The consequences of Zn deficiency are profound and far-reaching, ranging from increased individual morbidity and mortality to problems with community and regional economic development [4].

Attempts to alleviate the problem of Zn deficiency, both in Pakistan and in other countries, have followed several strategies. Providing micronutrient supplements, usually using "sprinkles", has been attempted under different circumstances, generally with some success [5]. However, it is an expensive and intensive approach, and without close supervision it can often be that the intended target, for example a pregnant woman, does not end up receiving the supplement. Fortification of foods at the processing stage has also been widely adopted, but there are significant problems with compliance and efficacy, both at the fortification stage where quality control is ineffective, and at the level of consumer uptake. A major issue is that poorer households in rural areas of developing countries typically grow the majority of their food and process it at village or community level. Thus, cereal fortification at large-scale processing facilities may not reach poorer households, where micronutrient deficiencies are often more prevalent and severe.

An alternative approach is to intervene at agricultural production stage through biofortification to achieve greater concentrations of bioavailable vitamins or elements in the edible portion of crops [6]. Biofortification can be realised through crop breeding or application of element-enriched fertilisers, known as "agronomic biofortification", which is particularly important in Pakistan where plant-available soil Zn concentrations are low [7]. Such Zn deficient soils that constitute 52% at national scale are also prevalent in Peshawar, Khyber Pakhtunkhwa Province, where plant-available soil Zn concentrations are significantly below the threshold 1 mg/kg in 75% of the soils analysed. Farmers in Pakistan rarely apply Zn fertilizers to their crops except for rice. Biofortification approaches have focused on staple crops because they are widely consumed including households with low purchasing power. Recently, varieties of maize, pearl millet and beans with high iron (Fe) and Zn concentrations

For peer review only - http://bmjopen.bmj.com/site/about/guidelines.xhtml

have been developed by the HarvestPlus programme for deployment in low-income settings. Human feeding studies have demonstrated that such varieties can lead to improvements in Fe and Zn intake and status [8,9], although there may be issues with low bioavailability [10]. Agronomic biofortification has not previously been deployed in a low-income setting but does have policy precedent in Finland, where granular fertilizers were enriched with selenium (Se) since the mid 1980's with consequent increases in human dietary Se intakes and eventual elimination of Se deficiency as a public health problem [11].

Preliminary studies have shown that Zn concentrations in wheat can be raised substantially through biofortification strategies[12]. It is notable that increasing levels of nutrients in the whole plant is not sufficient; elements must be translocated to the edible component of the grain (endosperm) and must be bioavailable. Phytic acid (PA) is the most important inhibitor of Zn absorption from wheat, therefore the Zn:PA molar ratio of the endosperm is an important consideration. The process of element accumulation in the endosperm is not simple. For example, Zn moves through many different transport processes prior to being deposited in the seed [13]. In a recent set of experiments within the HarvestPlus program over a period of eight years, scientists in Pakistan using traditional breeding techniques have been able to generate a "high Zn" variety of wheat that is adapted to Pakistan conditions. The variety (Zincol-2016/NR-421) contains more Zn per kg of grain compared to traditional varieties. However, grain Zn concentration will also depend on the availability of Zn in the soil, and there are likely to be synergistic effects of breeding for high Zn with application of Zn fertilizers.

The efficacy of increasing dietary intake through biofortification on individual and population Zn status is notoriously difficult to measure. The assessment of Zn status has been the subject of considerable research and is summarised comprehensively in two recent reviews supported by multinational expert groups [14,15]. Both reviews conclude that plasma or serum Zn concentration, the most widely used biomarker, is useful provided that various confounding factors are considered (including concurrent infection and inflammation). However, the plasma/serum must be collected following stringent methodology to avoid contamination and can be difficult to achieve in field settings. Hair Zn concentration is also potentially useful, and samples are easy to collect and store. In addition, emerging techniques, including the measurement of DNA fragmentation using the Comet assay and analysis of nail Zn concentration using laser ablation, are gaining credibility as useful biomarkers of Zn status. However, field trials are needed to evaluate their usefulness.

The primary aim of this study is to measure the impact of consuming flour made from the recently released high Zn wheat grain variety Zincol-2016/NR-421 on dietary Zn intake and

For peer review only - http://bmjopen.bmj.com/site/about/guidelines.xhtml

biomarkers of Zn status, in a low resource rural community setting in Pakistan. The secondary aim is to evaluate the potential usefulness of new biomarkers of Zn status. These aims will be achieved through conducting a randomised controlled feeding study with a crossover design. Comparison of outcome measures will be made following consumption of flour made from the biofortified wheat variety, Zincol-2016/NR-421, and the control, non-biofortified variety, Galaxy. The trial has been registered with the ISRCTN registry, study ID ISRCTN83678069 [16].

METHODS AND ANALYSIS

Study Setting and recruitment.

This study was conducted from the Health Centre that serves a community of approximately 5000 households in an area of 8 km^2 on the outskirts of Peshawar in Khyber Pakhtunkhwa (KPK). This community was chosen because previous studies have indicated that Zn deficiency is widespread, the diet is limited and primarily vegetable-based (thus low in bioavailable Zn) and the staple is wheat which is used for making chapatti and roti[17]. The catchment area, known as a Union Council, is made up of 10 villages (clusters). Out of 10 clusters in the union council, 5 were randomly selected-, from which equal number of households were randomly selected for the study. Selection of these clusters and households into intervention or control arm was done using a block design. Water samples were collected in duplicate from four different communal tube wells (hand pump) supplying each village, and analysed for mineral content by inductively coupled plasma-mass spectrometry (ICP-MS). The mean Zn concentration of the water from one cluster was high enough to significantly impact on daily Zn intake (>250 μg/L) therefore this cluster was eliminated from the study and an alternative cluster was randomly selected. The mean Zn concentration in the water analysed from the five included clusters was ≤120 μg/L and therefore not considered to add significantly to the daily Zn intake, based on a consumption of 2-4 L water per day (<0.5 mg Zn per day).

Ten households from each cluster were randomly selected. The head of the household was approached, the purpose of the study explained and, if eligible, the family invited to participate in the trial. If the head of the household declined, another house from the cluster was randomly selected and the invitation process repeated. Inclusion criteria were: the household included a female aged 16-49 years who was neither pregnant nor breastfeeding, and not currently consuming nutritional supplements. When there was more than 1 eligible woman in the household, one was conveniently selected with agreement from the head of the household. There were no additional exclusion criteria. Following informed consent, each household was assigned to treatment arm A or B (refer to figure 1) using a block design. A

For peer review only - http://bmjopen.bmj.com/site/about/guidelines.xhtml

sample size of 40, 95% significance level (2-sided) and 90% power, will enable an increase in plasma Zn concentration of 3.1 μg/dL to be detected. The target was to recruit 50 households (50 eligible women) to be able to accommodate a 20% attrition rate during the 18-week study. The project manager led the recruitment process because he is known to the community through previous health-related projects associated with the Abaseen Foundation PK (AFPK), a local NGO and key member of this project team, along with its sister organisation Abaseen Foundation UK (AFUK). There is a high level of trust and cooperation between community members and the AFPK who operate the Health Centre that serves this Union Council, therefore high levels of participant enrolment has been achieved. The project manager was responsible for allocating participants to each arm of the crossover trial using a block design. Only the project manager had access to this information.

The participants and the remaining research team members, including the PI and CoPIs, database manager and data analysts were blind to the order in which households received the biofortified or control flour. Unblinding was permissible if any adverse reactions are reported by the households during the trial.

Study design

We have conducted a randomised controlled feeding study with crossover design in which households were provided with flour milled from genetically and agronomically biofortified grain (Zincol-2016/NR-421) or control grain (Galaxy-2013). Galaxy-2013 was chosen for the control grain because it is widely cultivated across the country and is popular among farmers due to its higher yield. It did not receive Zn-containing fertilizers. Zincol-2016 (formerly known as NR-421 strain/line) was bred by the National Agriculture Research System, Pakistan with the support of HarvestPlus and approved by the Punjab Seed Council, Pakistan in 2016.[18] The grain for both the genotypes was sown at a farm in District Vehari District, Punjab, Pakistan in November 2016, and harvested in May 2017 by our project partner, Fauji Fertilizer Company (FFC) Limited. During the growing period, Zn-enriched fertilizer (Zn 13% as EDTA Zn) was applied to Zincol-2016 genotype at 1.25 kg/ha through injection into the irrigation system. This was followed by five foliar sprays of Zn ($ZnSO_4 \cdot H_2O$ 33% Zn) applied at 1 kg/ha of product dissolved in 250 L of water and sprayed per hectare at the booting stage. Analyses revealed that the Zn concentration in grains were 62 (SD 7.7) mg/kg and 27 (SD 4.3) mg/kg for the biofortified and control grain respectively. The grain was transported from the farm to the study field site in clearly labelled sacks (control and biofortified). The bags of grain were manually cleaned (hand removal of debris, stones, stalks), mixed and milled at a local facility. Care was taken to avoid cross contamination from

For peer review only - http://bmjopen.bmj.com/site/about/guidelines.xhtml

previously milled grain. Samples of flour from each sack (top, middle and bottom) were retained for analysis.

The intervention trial was comprised of a 2-week baseline equilibration period and two 8-week intervention/control periods. The baseline period started on 1st October 2017. During the baseline period, all the participating households were provided with control flour. After two weeks, Group A received biofortified flour and Group B received control flour. After 8 weeks, the two groups will cross over, with Group A receiving control flour and Group B receiving biofortified flour for the final 8 weeks (Figure 1). Freshly milled flour was be provided on a fortnightly basis for consumption by the entire household (average seven individuals, including children). Families will be asked not to consume any other flour within the household during the 18-week study period. Compliance was monitored at each flour distribution time point through interview with the head of the household. The number of meals consumed outside the household by the primary study participant (woman of child-bearing age) was recorded. Our previous knowledge of this community indicates that, other than community events such as weddings, it is unusual for women to eat outside the home.

Figure 1: Overview of Objective 1

Primary outcome measures

Flour is used primarily for making roti and chapatti in this community. The contribution of the total Zn intake from foods made from flour will be determined using 24-hour recall, coupled with food composition databases (e.g. WinDiets 2005® Research software, Robert Gordon University, UK). In addition, a selection of composite meal samples will be analysed. The impact of consuming the biofortified flour on the Zn status of women of childbearing age will be assessed by measuring plasma Zn concentration on ICP-MS analysis, DNA fragmentation by comet assay, and hair and nail Zn concentration by ICP-MS. In addition, we will measure concentrations of inflammatory biomarkers C-reactive protein (CRP) and alpha 1-acid glycoprotein that will be used to adjust plasma Zn concentration if required [19].

Dietary Analysis

Dietary nutrient intake will be assessed by analysing the 24-hour recalls, that were collected by interviewing the women participants by the study Nutritionist, who has local knowledge of the foods consumed and speaks Pushto, the local language. From our previous research[17] we know that the local diet is very limited in variety and monotonous and therefore a total of five recall interviews, taken at each of the sampling points (Figure 1), will give a good estimate of typical nutrient intake. Portion sizes will be estimated using household

For peer review only - http://bmjopen.bmj.com/site/about/guidelines.xhtml

measures, using items that are familiar to the households (e.g. plates, cups, spoons). Macro and micronutrient composition, plus phytate will be calculated from appropriate food composition databases. In addition, a selection of commonly consumed composite meals will be analysed for mineral, macronutrient and phytic acid concentration. During this interview, the nutritionist also collected data regarding adverse health-related events since the last measurement time point. Mothers were also asked to report on the frequency and duration of diarrhoeal episodes amongst children in the household. Any major concerns were reported to the Health Centre Doctor for follow up.

Biomarkers of Zn status

Tissue samples were collected from participants attending the Health Centre at five time points: baseline, mid and end of intervention phase 1 and mid and end of phase 2 (Figure 1).

Plasma Zn concentration

Whole blood was collected into trace element free tubes (BD Diagnostics, Switzerland) and the plasma separated by centrifugation within 40 minutes. Plasma 300 µl aliquots were transferred into Eppendorf tubes and stored at -20°C. Plasma samples will be shipped on dry ice to the University of Nottingham for analysis by ICP-MS.

DNA fragmentation analysis

DNA fragmentation (measured using the Comet assay) has been shown to decrease following dietary Zn supplementation [20]. Whole blood was collected by venous blood draw. Duplicate samples of 50 µl of whole blood was mixed with 50 µl cryopreservation buffer (85% RPMI, 15% DMSO) in a cryovial, and stored in a cryopreservation container in a fridge for up to 8 hours. Samples were transferred from the health centre to KMU on ice and placed in a -80°C freezer overnight (up to 12 hours), then transferred to liquid nitrogen for storage until samples at all five time points have been collected. DNA fragmentation will be quantified using the method of Singh et al [21 22], and Comet Assay IV Lite software (Perceptive Instruments, London, UK) for tail moment measurement.

Nail and hair analysis

Whole hairs were collected by combing through the hair and collecting individual hairs from the comb, making sure that the tag (cells from follicle) is attached to the proximal end of the hair. Nail samples were collected at baseline. Nail samples will be collected two more times at approximately 7 months (average time taken for a nail to grow from formation in the nail bed to the tip of the finger) from the start of phase 1 and phase 2. The Zn concentration of

For peer review only - http://bmjopen.bmj.com/site/about/guidelines.xhtml

hair and nail samples will be determined using laser induced breakdown spectroscopy[23] at NeurobioTex (Galveston, Texas, USA).

Additional outcomes

Anthropometry

Height and weight of the women was measured at baseline, and weight was monitored at each sample collection time point. Height was measured using a height board. The participant was to stand still with her back towards the scale, with the heels, back of the calves, upper back and the back of their head touching the board with the scale. The head was positioned so that the Frankfurt line was straight. The measuring device was lowered gently on the hair until it rested gently on the scalp. For the measurement of weight, women were asked to relax for 5 minutes before being asked to remove added clothes, shoes, socks, anything in the pocket, watch or necklace. The woman was asked to stand on the marked position on the scale until the digital reading or the reading needle stabilized. For consistency, weight was measured three times and the average calculated. In addition, the height and weight of at least one child under ten years in each household will be monitored throughout the intervention period. Mothers were also be asked to keep a diary of the frequency and duration of diarrhoeal episodes of children in the household throughout the intervention period.

Haematological and Micronutrient analyses

Haematocrit, haemoglobin, mean corpuscular volume (MCV), total iron binding capacity (TIBC), transferrin saturation will be estimated using clinical haematology methods. Blood samples will be analysed for micronutrient status including serum transferrin receptor and ferritin (sandwich ELISA), Fe and Se concentrations (ICP-MS), vitamin A (retinol binding protein (RBP)), and inflammatory markers (α1-acid glycoprotein (AGP) and C- reactive protein (CRP)).

Dark adaptation

The potential impact of increasing dietary Zn intake on dark adaptation was measured at baseline week 2, week 10 and week 18 during the participants' visits to the Health Centre. Dark adaptation was measured using a novel device developed by Palmer and co-workers[24].

Stool samples for parasite infection and microbiome analysis

Stools were collected at baseline week 2, week 10 and week 18. Samples were analysed within 24 hours for presence of parasites. Samples were stored to create a biobank for future short chain fatty acid and microbiome analysis.

For peer review only - http://bmjopen.bmj.com/site/about/guidelines.xhtml

DATA COLLECTION AND STORAGE

Sample analysis will take place in a number of different specialist laboratories in the UK (University of Nottingham: plasma Zn, Fe and Se, water, flour), USA (NeurobioTex: nail and hair Zn concentration) and Pakistan (Khyber Medical University: comet assay, haematology, serum transferring receptor, ferritin, RBP, and inflammatory markers). Comet assay tail moment analysis will be undertaken by both PK (KMU) and UK (University of Central Lancashire) based teams, and the results compared. Any significant differences will be resolved with expert support from the Children's Hospital Oakland Research Institute (CHORI). Sample analysis will be undertaken in duplicate as a minimum requirement. Data entry will be undertaken by the expert laboratory, and encrypted spreadsheets sent to the data hub where a final check for anomalies will be made by the database manager. Any potential anomalies identified will be checked with the expert lab. All data will be managed compliant with standard BBSRC policies http://www.bbsrc.ac.uk/web/FILES/Policies/data-sharing-policy.pdf. The University of Central Lancashire (UCLan) will be the data hub for the project; UCLan adheres to an Open Data policy and has an open data repository. Metadata will be collected in the form of "read me" files using basic Dublin Core. This study will generate small quantities of personal data from the analysis of blood and hair/nail samples. Most of the participants were illiterate so verbal informed consent was obtained and recorded by the project manager. Participants were given an ID number on recruitment, and this will be used in all data files. The list of names associated with the ID numbers is known only by the project manager, and is stored in a locked filing cabinet. Data collected in the field on laptops is password protected. UCLan systems are automatically backed up. Transfer of data between laboratories will be in the form of encrypted excel or word files. Data and metadata will be securely stored in a project database and reported on publication. Data will be shared with the project steering committee during the bi-monthly meetings. This committee is comprised of the PI and Co-Is. The database manager is undertaking the role of data monitoring and all members will have access to the final database.

STATISTICAL ANALYSIS PLAN

The analysis will be performed as intention to treat. Descriptive statistics will summarise participants' characteristics and outcome measurements at each time point, using methods appropriate for the type of data. Most study outcome measurements will be quantitative data. The primary outcome, plasma Zn concentration, was collected at 5 time points (baseline, week 4, 8, 12 and 16). After adjustment for inflammatory markers and as described above, the primary outcome will be assessed in two type comparisons:

I. Within group (A and B) comparisons.

For peer review only - http://bmjopen.bmj.com/site/about/guidelines.xhtml

Within each group of participants, the study outcome will be compared at appropriate time point (baseline will be compared with week 4 and week 8, week 4 compared with week 8, week 12 with week 16) to assess trend of the change (increase or decrease) in Zn concentrations. The pairwise comparisons will be done using paired t-test or Wilcoxon signed rank test if normality assumptions for t-test are not satisfied. Repeated measures ANOVA (or its non-parametric analogue) will be applied for comparisons at multiple time points (e.g. to compare study outcome at baseline, week 4 and week 8).

II. Comparisons between group A and B.

To answer the primary research question of determining the effectiveness of consuming flour made from agronomically biofortified wheat for improving Zn status, study outcomes will be compared between group A and B at week 8 and week 16, complemented with comparisons at interim collection points, week 4 and week 12. The pairwise comparisons will be done using independent samples t-test or Mann-Whitney U test as appropriate. Linear mixed modelling will be used for adjusted analyses to account for the baseline, repeated measurements and relevant covariates. Similar analysis will be done for all secondary outcomes measuring Zn status.

ETHICS AND DISSEMINATION

Ethical approval for the research has been granted from the lead University (UCLan) (unique reference number: STEMH 697 FR) and the collaborating institution in Pakistan, Khyber Medical University. All data collected will be anonymized and data protection protocols followed when information is shared between participating institutions.

The final study methods (including any modifications) will be published in peer reviewed journals, alongside the study outcomes on completion of the data analysis. Authorship will be restricted to those who have contributed to the design of the study, data collection and analysis, in accordance with journal guidelines. In addition to peer reviewed journals, findings will be disseminated to the scientific community via conference presentations and abstracts. The findings of the study will be communicated to the study participants through the village elders at an appropriate community forum. More widely, the AFPK can access some of the most difficult to reach communities in KPK and the Federally Administered Tribal Area (FATA), including through tribal networks, local Jirga and religious leaders. We will work with our contacts in the agriculture sector to exchange knowledge with key actors. For example, the Agricultural Services Department of FFC provides soil, water, fertilizer, and plant analysis and recommendation services (in addition to other farmer education/training

For peer review only - http://bmjopen.bmj.com/site/about/guidelines.xhtml

programmes) to the farming community throughout the country. Project partners and collaborators are well connected to ensure that new evidence can support policy.

AUTHORS CONTRIBUTIONS

Nicola Lowe, Martin Broadley, Munir Zia, Harry McArdle, Jaffar Khan, Edward Joy, and Mukhtiar Zaman conceived the research questions and designed the study protocol. Nicola Lowe is the principal investigator, Martin Broadley, Jaffar Khan, Harry McArdle, Edward Joy, Munir Zia, and Mukhtiar Zaman are co-investigators. Rashid Medhi is managing the community engagement and recruitment, Babar Shahzad, Ubaid Ullah and Gul Khubana are conducting the field work and sample collection. Heather Ohly was involved in the original conception of the study, and is managing the study database at the project hub (UCLan). All authors have read and approved this manuscript.

FUNDING

This work is supported by BBSRC Global Challenges Research Fund, Foundation Awards for Global Agriculture and Food Systems Research. Grant Number BB/P02338X/1

We are grateful for the in-kind contribution of Fauji Fertilizer Company (FFC) Limited for crop management and production, and to HarvestPlus for supplying the Zincol-2016/NR-421 wheat grain for sowing.

We would also like to acknowledge the in-kind contribution of Professor Janet King and Dr. Swapna Shenvi at the Children's Hospital Oakland Research Institute (CHORI) for support with the Comet assay, and Dr Amanda Palmer and Dr Katie Healy at Johns Hopkins University for training and loan of the dark adaptation detection equipment.

The authors gratefully acknowledge the Abaseen Foundation, Pakistan for use of the Health Centre and facilitating access to the community. Also Dr Sveltlana Tishkovskaya at UCLan for Statistical support.

CONFLICTS OF INTEREST

The authors report no conflicts of interest.

REFERENCES

1. Wessells KR, Brown KH. Estimating the Global Prevalence of Zinc Deficiency: Results Based on Zinc Availability in National Food Supplies and the Prevalence of Stunting. *Plos One* 2012;7(11)

2. Kumssa DB, Joy EJM, Ander EL, et al. Dietary calcium and zinc deficiency risks are decreasing but remain prevalent. *Sci Rep-Uk* 2015;5

For peer review only - http://bmjopen.bmj.com/site/about/guidelines.xhtml

3. Agha Khan University. Planning Commission. Planning and Development Division. Government of Pakistan. National Nutrition Survey Pakistan 2011.

4. Boerma JT, Bryce J, et al. Mind the gap: equity and trends in coverage of maternal, newborn, and child health services in 54 Countdown countries. *Lancet* 2008;371(9620):1259-67.

5. Allen LH, Peerson JM, Olney DK. Provision of multiple rather than two or fewer micronutrients more effectively improves growth and other outcomes in micronutrient-deficient children and adults. *J Nutr* 2009;139(5):1022-30.

6. Varo P, Alfthan G, Ekholm P, et al. Selenium intake and serum selenium in Finland: effects of soil fertilization with selenium. *Am J Clin Nutr* 1988;48(2):324-9.

7. Edward J. M. Joy WA, Munir H. Zia, Diriba B. Kumssa, Scott D. Young, E. Louise Ander, Michael J. Watts, Alexander J. Stein, Martin R. Broadley. Valuing increased zinc (Zn) fertiliser-use in Pakistan. *Plant and Soil* 2016.

8. Cercamondi CI, Egli IM, Mitchikpe E, et al. Total iron absorption by young women from iron-biofortified pearl millet composite meals is double that from regular millet meals but less than that from post-harvest iron-fortified millet meals. *J Nutr* 2013;143(9):1376-82.

9. Chomba E, Westcott CM, Westcott JE, et al. Zinc absorption from biofortified maize meets the requirements of young rural Zambian children. *The Journal of nutrition* 2015;145(3):514-9.

10. Petry N, Egli I, Gahutu JB, et al. Stable iron isotope studies in Rwandese women indicate that the common bean has limited potential as a vehicle for iron biofortification. *J Nutr* 2012;142(3):492-7.

11. Alfthan G, Eurola M, Ekholm P, et al. Effects of nationwide addition of selenium to fertilizers on foods, and animal and human health in Finland: From deficiency to optimal selenium status of the population. *JTEMB* 2015;31:142-47.

12. Coralie Signorell DM, Christophe Zeder, Fabian Tay, Erick Boy, Neelam Khetarpaul, Ismail Cakmak, Michael Zimmermann. Evaluation of Zinc Bioavailability in Humans from Foliar Zinc Biofortified Wheat and from Intrinsic vs. Extrinsic Zn Labels in Biofortified Wheat. *Eur J Nutr & Food Safety* 2015;5(5):863-64.

13. Olsen LI, Palmgren MG. Many rivers to cross: the journey of zinc from soil to seed. *Frontiers in PLand Sci* 2014;5:1-6.

14. King JC, Gibson RS, Krebs NF, Lowe NM, Siekmann JH, Raiten DJ. Biomarkers of Nutrition for Development (BOND)-Zinc Review. *J Nutr* 2016.

15. Lowe NM, Fekete K, Decsi T. Methods of assessment of zinc status in humans: a systematic review. *Am J Clin Nutr* 2009;89(6):2040s-51s.

For peer review only - http://bmjopen.bmj.com/site/about/guidelines.xhtml

16. ISRCTN83678069. Biofortification with zinc in flour for eliminating deficiency: BioMed Central; 2017 [Available from: https://www.isrctn.com/ISRCTN83678069 accessed 30/11/2017].

17. Khan MJ, Usama UU, Lowe N, Broadley M, Afridi MZ, Zia M, McArdle HM, Young S. Effect of agronomically biofortified zinc flour on zinc and selenium status in resource poor settings; a randomised control trial. *Proc Nutr Soc* 2017;76(OCE4).

18. Singh RaG, Velu. Zinc-Biofortified Wheat: Harnessing Genetic Diversity for Improved Nutritional Quality. *Science Brief: Biofortification No 1 (May 2017) CIMMYT, HarvestPlus, and the Global Crop Diversity Trust Bonn, Germany*.

19. Raiten DJ, Sakr Ashour FA, Ross AC, et al. Inflammation and Nutritional Science for Programs/Policies and Interpretation of Research Evidence (INSPIRE). *J Nutr* 2015;145(5):1039S-108S.

20. Joray ML, Yu T-W, Ho E, et al. Zinc supplementation reduced DNA breaks in Ethiopian women. *Nutr Res* 2015;35(1):49-55.

21. Singh NP, McCoy MT, Tice RR, et al. A simple technique for quantitation of low levels of DNA damage in individual cells. *Exp Cell Res* 1988;175(1):184-91.

22. Zyba SJ, Shenvi SV, Killilea DW, et al. A moderate increase in dietary zinc reduces DNA strand breaks in leukocytes and alters plasma proteins without changing plasma zinc concentrations. *Am J Clin Nutr* 2017;105(2):343-51.

23. Riberdy VA, Frederickson CJ, Rehse SJ. Determination of the Zinc Concentration in Human Fingernails Using Laser-Induced Breakdown Spectroscopy. *App Spectr* 2017;71(4):567-82.

24. Labrique AB, Palmer AC, Healy K, et al. A novel device for assessing dark adaptation in field settings. *BMC Ophthalmol* 2015;15:74.

For peer review only - http://bmjopen.bmj.com/site/about/guidelines.xhtml

Figure Legend

Figure 1: Schematic overview of objective 1, randomised controlled feeding study with crossover design.

For peer review only

For peer review only - http://bmjopen.bmj.com/site/about/guidelines.xhtml

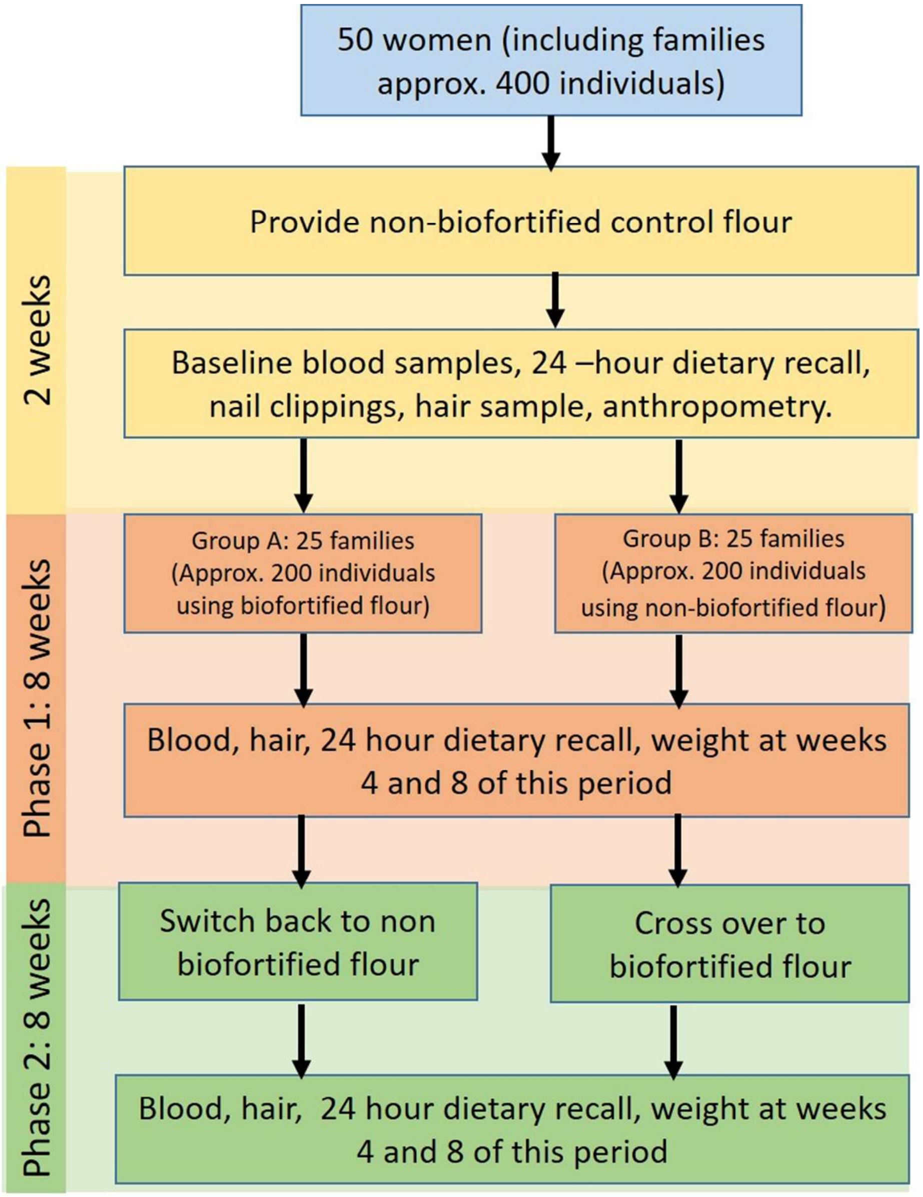

Figure 1

71x93mm (300 x 300 DPI)

STANDARD PROTOCOL ITEMS: RECOMMENDATIONS FOR INTERVENTIONAL TRIALS

SPIRIT 2013 Checklist: Recommended items to address in a clinical trial protocol and related documents*

Section/item	Item No	Description	Addressed on page number
**Administrative information**			
Title	1	Descriptive title identifying the study design, population, interventions, and, if applicable, trial acronym	_____1________
Trial registration	2a	Trial identifier and registry name. If not yet registered, name of intended registry	_____1________
	2b	All items from the World Health Organization Trial Registration Data Set	___yes_______
Protocol version	3	Date and version identifier	_______N/A___
Funding	4	Sources and types of financial, material, and other support	____11_____
Roles and responsibilities	5a	Names, affiliations, and roles of protocol contributors	12 & cover page
	5b	Name and contact information for the trial sponsor	____N/A_____
	5c	Role of study sponsor and funders, if any, in study design; collection, management, analysis, and interpretation of data; writing of the report; and the decision to submit the report for publication, including whether they will have ultimate authority over any of these activities	_____N/A_______
	5d	Composition, roles, and responsibilities of the coordinating centre, steering committee, endpoint adjudication committee, data management team, and other individuals or groups overseeing the trial, if applicable (see Item 21a for data monitoring committee)	___12____

For peer review only - http://bmjopen.bmj.com/site/about/guidelines.xhtml

**Introduction**			
Background and rationale	6a	Description of research question and justification for undertaking the trial, including summary of relevant studies (published and unpublished) examining benefits and harms for each intervention	__2-4__
	6b	Explanation for choice of comparators	__5_______
Objectives	7	Specific objectives or hypotheses	_______4_______
Trial design	8	Description of trial design including type of trial (eg, parallel group, crossover, factorial, single group), allocation ratio, and framework (eg, superiority, equivalence, noninferiority, exploratory)	__5-7______
**Methods: Participants, interventions, and outcomes**			
Study setting	9	Description of study settings (eg, community clinic, academic hospital) and list of countries where data will be collected. Reference to where list of study sites can be obtained	__4,5_____
Eligibility criteria	10	Inclusion and exclusion criteria for participants. If applicable, eligibility criteria for study centres and individuals who will perform the interventions (eg, surgeons, psychotherapists)	_4,5___
Interventions	11a	Interventions for each group with sufficient detail to allow replication, including how and when they will be administered	__4,5_________
	11b	Criteria for discontinuing or modifying allocated interventions for a given trial participant (eg, drug dose change in response to harms, participant request, or improving/worsening disease)	_____N/A______
	11c	Strategies to improve adherence to intervention protocols, and any procedures for monitoring adherence (eg, drug tablet return, laboratory tests)	____6_______
	11d	Relevant concomitant care and interventions that are permitted or prohibited during the trial	___N/A______
Outcomes	12	Primary, secondary, and other outcomes, including the specific measurement variable (eg, systolic blood pressure), analysis metric (eg, change from baseline, final value, time to event), method of aggregation (eg, median, proportion), and time point for each outcome. Explanation of the clinical relevance of chosen efficacy and harm outcomes is strongly recommended	____7-9______
Participant timeline	13	Time schedule of enrolment, interventions (including any run-ins and washouts), assessments, and visits for participants. A schematic diagram is highly recommended (see Figure)	_7 (FIGURE 1)

For peer review only - http://bmjopen.bmj.com/site/about/guidelines.xhtml

Sample size	14	Estimated number of participants needed to achieve study objectives and how it was determined, including clinical and statistical assumptions supporting any sample size calculations	___5__________
Recruitment	15	Strategies for achieving adequate participant enrolment to reach target sample size	____4________

Methods: Assignment of interventions (for controlled trials)

Allocation:			
Sequence generation	16a	Method of generating the allocation sequence (eg, computer-generated random numbers), and list of any factors for stratification. To reduce predictability of a random sequence, details of any planned restriction (eg, blocking) should be provided in a separate document that is unavailable to those who enrol participants or assign interventions	_____N/A_______ _
Allocation concealment mechanism	16b	Mechanism of implementing the allocation sequence (eg, central telephone; sequentially numbered, opaque, sealed envelopes), describing any steps to conceal the sequence until interventions are assigned	____N/A_______
Implementation	16c	Who will generate the allocation sequence, who will enrol participants, and who will assign participants to interventions	____5_______
Blinding (masking)	17a	Who will be blinded after assignment to interventions (eg, trial participants, care providers, outcome assessors, data analysts), and how	____5________
	17b	If blinded, circumstances under which unblinding is permissible, and procedure for revealing a participant's allocated intervention during the trial	___5_______

Methods: Data collection, management, and analysis

Data collection methods	18a	Plans for assessment and collection of outcome, baseline, and other trial data, including any related processes to promote data quality (eg, duplicate measurements, training of assessors) and a description of study instruments (eg, questionnaires, laboratory tests) along with their reliability and validity, if known. Reference to where data collection forms can be found, if not in the protocol	___7-10______
	18b	Plans to promote participant retention and complete follow-up, including list of any outcome data to be collected for participants who discontinue or deviate from intervention protocols	___10______

For peer review only - http://bmjopen.bmj.com/site/about/guidelines.xhtml

Data management	19	Plans for data entry, coding, security, and storage, including any related processes to promote data quality (eg, double data entry; range checks for data values). Reference to where details of data management procedures can be found, if not in the protocol	___10_______
Statistical methods	20a	Statistical methods for analysing primary and secondary outcomes. Reference to where other details of the statistical analysis plan can be found, if not in the protocol	__10-11____
	20b	Methods for any additional analyses (eg, subgroup and adjusted analyses)	____N/A______
	20c	Definition of analysis population relating to protocol non-adherence (eg, as randomised analysis), and any statistical methods to handle missing data (eg, multiple imputation)	__N/A______
**Methods: Monitoring**			
Data monitoring	21a	Composition of data monitoring committee (DMC); summary of its role and reporting structure; statement of whether it is independent from the sponsor and competing interests; and reference to where further details about its charter can be found, if not in the protocol. Alternatively, an explanation of why a DMC is not needed	_____10_____
	21b	Description of any interim analyses and stopping guidelines, including who will have access to these interim results and make the final decision to terminate the trial	___N/A______
Harms	22	Plans for collecting, assessing, reporting, and managing solicited and spontaneously reported adverse events and other unintended effects of trial interventions or trial conduct	_______8___
Auditing	23	Frequency and procedures for auditing trial conduct, if any, and whether the process will be independent from investigators and the sponsor	___N/A_______
**Ethics and dissemination**			
Research ethics approval	24	Plans for seeking research ethics committee/institutional review board (REC/IRB) approval	____11_____
Protocol amendments	25	Plans for communicating important protocol modifications (eg, changes to eligibility criteria, outcomes, analyses) to relevant parties (eg, investigators, REC/IRBs, trial participants, trial registries, journals, regulators)	_____11_____

For peer review only - http://bmjopen.bmj.com/site/about/guidelines.xhtml

Consent or assent	26a	Who will obtain informed consent or assent from potential trial participants or authorised surrogates, and how (see Item 32)	____5_______
	26b	Additional consent provisions for collection and use of participant data and biological specimens in ancillary studies, if applicable	___N/A_______
Confidentiality	27	How personal information about potential and enrolled participants will be collected, shared, and maintained in order to protect confidentiality before, during, and after the trial	________10___
Declaration of interests	28	Financial and other competing interests for principal investigators for the overall trial and each study site	______12_____
Access to data	29	Statement of who will have access to the final trial dataset, and disclosure of contractual agreements that limit such access for investigators	___10_______
Ancillary and post-trial care	30	Provisions, if any, for ancillary and post-trial care, and for compensation to those who suffer harm from trial participation	__N/A_________
Dissemination policy	31a	Plans for investigators and sponsor to communicate trial results to participants, healthcare professionals, the public, and other relevant groups (eg, via publication, reporting in results databases, or other data sharing arrangements), including any publication restrictions	____11_______
	31b	Authorship eligibility guidelines and any intended use of professional writers	______12_____
	31c	Plans, if any, for granting public access to the full protocol, participant-level dataset, and statistical code	____10_______
**Appendices**			
Informed consent materials	32	Model consent form and other related documentation given to participants and authorised surrogates	_appendix 1 and 2
Biological specimens	33	Plans for collection, laboratory evaluation, and storage of biological specimens for genetic or molecular analysis in the current trial and for future use in ancillary studies, if applicable	_9,10_______

*It is strongly recommended that this checklist be read in conjunction with the SPIRIT 2013 Explanation & Elaboration for important clarification on the items. Amendments to the protocol should be tracked and dated. The SPIRIT checklist is copyrighted by the SPIRIT Group under the Creative Commons "Attribution-NonCommercial-NoDerivs 3.0 Unported" license.

For peer review only - http://bmjopen.bmj.com/site/about/guidelines.xhtml
